# Effective Acoustic Model-Based Beamforming Training for Static and Dynamic Hri Applications

**DOI:** 10.3390/s24206644

**Published:** 2024-10-15

**Authors:** Alejandro Luzanto, Nicolás Bohmer, Rodrigo Mahu, Eduardo Alvarado, Richard M. Stern, Néstor Becerra Yoma

**Affiliations:** 1 Speech Processing and Transmission Laboratory, Electrical Engineering Department, University of Chile, Santiago 8370451, Chile; alejandro.luzanto@ug.uchile.cl (A.L.); njimenez.bohmer@gmail.com (N.B.); rmahus@gmail.com (R.M.); eduardo.alvarado@ug.uchile.cl (E.A.); nbecerra@ing.uchile.cl (N.B.Y.); 2 Department of Electrical and Computer Engineering, Carnegie Mellon University, Pittsburgh, PA 15213, USA

**Keywords:** voice-based user profiling, indoor acoustic model, static and dynamic HRI, deep learning-based beamforming, automatic speech recognition

## Abstract

Human–robot collaboration will play an important role in the fourth industrial revolution in applications related to hostile environments, mining, industry, forestry, education, natural disaster and defense. Effective collaboration requires robots to understand human intentions and tasks, which involves advanced user profiling. Voice-based communication, rich in complex information, is key to this. Beamforming, a technology that enhances speech signals, can help robots extract semantic, emotional, or health-related information from speech. This paper describes the implementation of a system that provides substantially improved signal-to-noise ratio (SNR) and speech recognition accuracy to a moving robotic platform for use in human–robot interaction (HRI) applications in static and dynamic contexts. This study focuses on training deep learning-based beamformers using acoustic model-based multi-style training with measured room impulse responses (RIRs). The results show that this approach outperforms training with simulated RIRs or matched measured RIRs, especially in dynamic conditions involving robot motion. The findings suggest that training with a broad range of measured RIRs is sufficient for effective HRI in various environments, making additional data recording or augmentation unnecessary. This research demonstrates that deep learning-based beamforming can significantly improve HRI performance, particularly in challenging acoustic environments, surpassing traditional beamforming methods.

## 1. Introduction

### 1.1. Human–Robot Collaboration

Human–robot collaboration has been a topic of great interest for research institutes, universities and companies in recent years, as it will play an important role in the fourth industrial revolution encompassing applications in hostile environments, mining, industry, forestry, education, natural disaster and defense [1]. Collaboration means working with someone on something in order to achieve a goal, which requires a common plan among those involved [2]. Ideally, the individuals involved should understand the intentions and tasks of the other party; this information is easy to obtain for the human but difficult to obtain for the robot. The most common forms of human–robot communication are voice, gestures, actions, haptic signals (joystick) and psychological signals, which can be very invasive for the user [3]. Voice (language) is a form of explicit communication that comes naturally to humans and contains a large amount of complex information, which can be difficult to extract by a machine or robot. In this sense, automatic speech recognition (ASR) systems play an important role [4,5] since they allow humans to use voice to communicate with computational interfaces, and, if a more complex analysis is necessary, they allow for the use of methods that process semantic information.

For the collaboration to be effective, the robot must be able to detect and profile the user. In this way, the robot is able to adapt and modify its behavior according to the profile of the target user, understanding its characteristics and preferences. In HRI situations, it is necessary to model and recognize human actions and capabilities in order to discover the intentions and objectives behind those actions. User characterization should be performed in three components: physical, cognitive, and social [6].

Physical: Characteristics related to the human body and movement in space are used. The profiling corresponds to the detection of movement capabilities related to the process of interaction with the movements provided in space.

Cognitive: It is necessary to detect and recognize the agent’s intentions [7]. For this purpose, the so-called theory of mind (ToM) is used, which seeks to infer and recognize the intentions, desires, personality and emotions [8] of the individuals [9].

Social: Robots must recognize and interpret a human’s social cues [10]. Social cues can be defined as observable behaviors that produce behavioral changes during interaction [11].

### 1.2. Voice-Based Human Interaction

Robust speech processing is critical to achieving such collaboration as well as meaningful profiling of humans. In other words, speech recognition, speech emotion recognition, prosody analysis and speech feature extraction need to be reliable and robust to additive noise and reverberation in both static and dynamic HRI conditions. The most natural form of human communication is the voice. The voice carries a large amount of linguistic and paralinguistic (prosodic) information that is of great use to the robot. The linguistic information is the message per se. Paralinguistic information is what accompanies the message (tone of voice, silences, intensity, etc.). In this sense, spoken language carries not only the message but also the accent, tone of voice, voice quality, social group, physical and psychological identity, mood, etc. [12]. With such information, the receiver understands the semantics of the message, and, with paralinguistic information, it is possible to extract the physical, cognitive and social state. Humans use this information to communicate and extrapolate the information received, which is difficult for the robot. Voice is becoming an essential medium of communication and interaction between computer agents and end users [13]. For the robot to be able to communicate by voice, it must be able to extract linguistic and paralinguistic content [14].

Linguistic content: The process of understanding semantic information is called semantic language understanding (SLU). Generally, a cascade procedure is used to extract this information, which consists of applying an ASR and then using natural language processing modules. Lately, approaches have been made using deep neural networks to extract semantic information directly from the signal [15].

Prosodic content: There are multiple approaches to extracting paralinguistic information. One of the best-known is to use signal features to describe characteristics that are used to profile the user. The use of prosodic features such as F0, duration, and voice quality has been proposed to evaluate and extract paralinguistic information such as intentions, attitudes, and emotions in a dialog [16]. Another very popular approach is the use of neural networks that can intrinsically extract paralinguistic information. The use of frameworks has been proposed to make neural networks learn paralinguistic information, which can be used in the task of emotion recognition [17].

In order to extract linguistic and paralinguistic content, the most important thing is to have good-quality acoustic signals. Signals with high degrees of noise and/or reverberation impair speech detection performance. For that reason, it is necessary to perform signal processing to separate the audio from the noise and/or reverberation to obtain an improved signal from which it is possible to extract such information.

Noise and reverberation are frequently present in real indoor applications that involve dynamic scenarios for the robot, such as movement and changes in the acoustical environment or task at hand; in order for the robot to perform properly in dynamic scenarios in real time, it must be able to perform speech enhancement under multiple conditions while in motion.

### 1.3. Beamforming

Beamforming is a powerful filtering technique that enables spatial discrimination and cancelation of the energy from interfering sources by combining the channels of a microphone array. Multiple methods have been proposed for combining the signals, of which the simplest and best known is delay-and-sum beamforming, which, as its name suggests, applies time delays to the channels in order to ensure that the signals from the direction of the desired target signal arrive in phase and reinforce each other. Signals arriving from other directions can interfere with each other destructively from channel to channel, which tends to reduce the energy of these interfering signals [18]. Other methods require the estimation of the target and noise covariance matrices, such as the minimum variance distortionless response (MVDR) beamformer [19] or the maximum signal-to-noise ratio (MSNR) beamformer [20], also known, by its solution, as a generalized eigenvalue beamformer (GEV) [21]. The first seeks to minimize the variance of the noise with the constraint of keeping the target signal undistorted, and the second seeks to maximize the SNR of the target signal. To estimate the covariance matrices, one of the techniques that has recently provided very good results in robust ASR challenges, such as CHiME [22,23], is the masking technique. This technique consists of finding time–frequency masks to isolate the sources based on the extent to which a given source is dominant in each time–frequency element in the signal spectrogram. There are different types of masks, among which the best known are the ideal binary masks (IBMs), which perform a hard decision assigning 1 or 0 if the frequency bin corresponds to the source, the ideal ratio masks (IRMs) [24,25], which perform a soft decision assigning values between [0 and 1] to the mask, and the complex ratio masks (CRMs), which are similar to IRMs but that also seek to recover the phase of the signal [26]. There are also extensions of the CRMs called complex ratio filters (CRFs) [27]. For mask estimation, there are statistical approaches using, for example, complex Gaussian mixture models (CGMM) [28] or approaches that make use of deep learning as fully-connected layers [29,30,31], recurrent neuronal networks (RNN) [32,33,34,35,36,37], UNet-based networks [38,39,40] and time convolutional networks (TCNs) [41], with the Conv-TasNet architecture being used in many beamforming schemes [42,43,44,45,46]. It is worth highlighting that beamforming methods carry out spatial filtering. Consequently, beamforming schemes help reduce the effect of reverberation by attenuating the acoustic signals received from directions other than the direct field of the target. Nevertheless, they do not attempt to de-reverberate directly acoustic signals that are recorded indoors.

Masking beamforming exploits both the ability of the time–frequency mask to isolate different sources to estimate the covariance matrices and the ability of spatial filtering to recover the signal arriving from the desired direction. After the masking process, it is necessary to compute the covariance matrices, for which there are different methods, such as in a time-invariant manner [29,33,34,35,42], online [28,36] or block-wise [47,48], and then obtain the weights by applying MVDR or GEV. Other schemes go further and compute the matrices using deep learning, such as [44], sometimes, even solving numerical stability problems that beamforming solutions have in operations such as matrix inversion [32,43], and, in the case of other methods, simply obtaining the beamforming weights directly using deep learning [45,46].

While many of these systems have been shown to perform well in both simulated and real static conditions, their behavior in real situations where the microphone array or sources move has not been explored very much. When changes in spatial conditions occur, it is necessary to adapt the covariances matrices over time. Also, the estimation of masks can be affected in methods that are based on spatial information, such as spatial clustering, [28] as opposed to methods that are based only on spectral information [29,34]. Other systems contain both explicit spectral and spatial information [33,43,45,46], but the impact of including this information in dynamic situations has not been studied. Some methods that have explored the dynamic case rely mainly on the adaptation of covariance matrices, where, in [44], the weights of the self-attention mechanisms are used to compute the covariance matrices. The CNN-LSTM architecture has been used to directly obtain three interpretations of the matrix adaptation [32]. Motion has also been addressed with the blockwise on-line processing of a mask-based beamformer [48]. However, these methods have been tested mostly in simulated dynamic conditions. Another way of dealing with dynamic situations is through source localization [49,50,51,52]; however, these methods depend on spatial characteristics extracted from the microphone array which are sensitive to motion, reverberation, noise and non-voice scenarios [53].

### 1.4. Training for Deep Learning-Based Beamforming

Generally, neural beamformers are trained and evaluated in a simulated multi-condition manner using multiple types of noise and room impulse responses (RIRs) generated with the image source method [54], which has been improved, as described in [55]. Nevertheless, with image-method simulations, it is difficult to estimate and incorporate all the effects of a real scenario, such as the exact geometry, materials and obstacles, and how exactly the sound propagates, especially at low frequencies. While real RIRs should be better than simulated ones to train masking or neural beamformers, this issue has not been studied exhaustively in the literature. In a similar problem, the use of simulated versus real RIRs in training deep neural networks (DNNs) for speech recognition has been explored [56], and it has been found, using existing datasets, that the use of real RIRs provides better results than the use of simulated RIRs. Recent studies have recognized the discrepancies between simulated and real RIRs, proposing various methods to improve the accuracy of simulated RIRs [57,58,59,60]. However, the comparison of results obtained using simulated and real RIRs has rarely taken place in the context of beamforming and microphone arrays, particularly in HRI scenarios. Furthermore, the use of multi-condition training of RIRs has not been addressed in the prior literature either. 

Neural beamforming, based on DNN mask estimators, has demonstrated good improvements in WER under real reverberant conditions [61]. The REVERB database, which contains utterances generated using simulated and real RIRs, is used in such studies, where the simulated RIRs are employed for training and the real RIRs are used for testing. It is shown that a DNN-based mask estimator with an analytic formulation to obtain a beamforming vector can achieve a significant WER reduction, going from 24.5% for unprocessed signals to 12.8% using neuronal beamforming. The REVERB dataset contains only static conditions, reverberated conditions, and those with very little noise. Therefore, training with these datasets may not be sufficient for more complex situations or situations involving motion.

Previous studies have also considered methods to enhance simulated RIRs in deep-learning models [62]. This involves the use of large-scale noisy data created by simulation of the RIR in order to increase accuracy in speech recognition. The results of testing the model in real conditions show that training with simulated RIRs reduces the WER compared to training with pseudo-clean utterances. Nevertheless, the quality of the trained model depends (unsurprisingly) on how good the simulation is and to what extent it captures the wide variety of use cases. A simulation is inevitably a simplification of reality; not all variables can be modeled.

When the problem of indoor reverberation has been addressed in multi-channel speech enhancement with measured RIRs, generally, these RIRs match the test conditions, or they are used only for performance-testing purposes. For example, in [63], a total of 120 measured RIRs are recorded in 24 fully furnished office rooms and augmented to 720 RIRs for training, in conjunction with another 720 simulated RIRs, using 640 of each for training and the rest for testing. Between the training and test sets, there may be RIRs from the same room but in different positions. In [64,65], measured RIRs were used to test the performance of multi-channel speech enhancement models, which were trained with simulated RIRs.

Alternatively, to cope with indoor reverberation in real scenarios, models can be trained with a combination of measured and simulated RIRs, as in the CHiME challenges [22,23], where real data were recorded in four scenarios in which two scenarios are recorded indoors and simulated data are generated by combining clean speech utterance with recorded noises from the four scenarios. This scheme of generating speech data in multiple scenarios can certainly converge to scenario-independent models. However, to carry out this task in such a way as to include a large number of speakers and scenarios is time-consuming and expensive.

Not surprisingly, the problem of indoor dynamic scenarios has been addressed mainly with simulated RIRs. In [32], a CNN-LSTM architecture was trained with dynamic data generated by computing static simulated impulse responses at 250 rotations and performing time-varying convolution. In [44], a masking beamformer was trained and evaluated with simulated RIRs that model a speaker moving in a straight line in a room while the microphone array and noise sources are fixed. Also, in [48], a voice activity detector (VAD) was trained with utterances generated using static simulated RIRs to apply beamforming techniques, where one of the test sets corresponded to a real dynamic utterance, which was mixed with four noises. One exception to this approach may be found in [66], where an entire dataset from a speaker walking in a room was recorded to train and test the performance of a deep neural network for source separation. In [67], measured RIRs were estimated and employed to train an ASR system that was tested with real static and dynamic data in the same room.

As previously mentioned, the use of measured RIRs to train deep learning-based beamformers has not been much explored, and, when they are used to train the beamformers, generally, they include part of the test conditions or are used only for performance-testing purposes. Furthermore, when these models have been studied to encompass dynamic situations, only simulated RIRs are used for training and evaluation. Because of this, a major objective of this paper is to compare the performance obtained by two masking beamformers when trained with both simulated and measured RIRs in real static and dynamic conditions. For our experiments, we use the Self-attentive MVDR beamformer (SA-MVDR) [44] and the Self-attentive RNN beamformer (SA-RNN) [45] because of their observed performance in pilot experiments compared to other state-of-the-art deep learning-based beamformers in the speaker separation task and their potential to handle scenarios that include motion. Specifically, we will evaluate the ability of multi-condition training using measured impulse responses to improve the efficacy of deep learning-based beamforming to cover static and dynamic real HRI scenarios. Additionally, the performance of neural beamforming is studied in comparison with more traditional approaches, such as delay-and-sum and MVDR beamforming using voice activity detection (VAD). In order to carry out this task, measured RIRs were measured in five rooms, one of which corresponds to the evaluation room. The test database is the same one that was used in [67], where a state-of-the-art robotic platform and a Personal Robot 2 (PR2) were used to record challenging utterances in static and dynamic conditions in an HRI context.

## 2. Traditional and Neural Beamforming Techniques

In this section, we briefly describe the traditional and neural beamformers used for our experiments.

### 2.1. Delay and Sum (D&S)

Delay-and-sum beamforming is one of the simplest and most commonly used techniques in traditional beamforming. As described above, D&S beamforming seeks to maximize the gains in the desired direction by applying time delays to the different signal channels to compensate for unequal distances between the target source and the microphones in the array. Since the PR2 robot records the rotation of its head, which corresponds to the Angle of Arrival (AOA) on the testbed, it is possible to calculate the different delays for both static and dynamic conditions. Using the AOA and assuming a plane wavefront, the time delays for an array of L microphones are defined by (1):

(1) $$\tau_{l}\;=\;\frac{\Delta_{l\;}\sin\left(\varphi \right)}{c}$$

where $\tau_{l}$ is the delay for the channel l, $\Delta_{l}$ is the distance between the reference channel and the channel l, φ  is the AOA and c corresponds to the wave propagation speed. 

### 2.2. Minimum Variance Distortionless Response (MVDR)

The MVDR beamformer is another widely used technology that is characterized by suppressing noise but keeping the desired signal undistorted [68]. For its implementation, the steering vector-based solution was used, which is presented by (2):

(2) $$w\left(\omega \right)=\frac{\Phi_{N}^{-1}\left(\omega \right)v\left(\omega \right)}{v^{H}\left(\omega \right)\Phi_{N}^{-1}\left(\omega \right)v\left(\omega \right)}$$

where $N\left(\omega \right)=\left[N_{1}\left(\omega \right)\;N_{2}\left(\omega \right)\ldots \;N_{l}\left(\omega \right)\ldots N_{L}\left(\omega \right)\;\right]$ denotes the spatially correlated noise in each microphone in the frequency domain, $\Phi_{N}\left(\omega \right)=\frac{1}{T}\sum_{t=1}^{T}N\left(w,t\right)N^{H}\left(w,t\right)$ is the noise covariance matrix and the vector $v\left(\omega \right)=\left[e^{-jw\tau_{0}}\;e^{-jw\tau_{1}}\;e^{-jw\tau_{2}}\;...\;e^{-jw\tau_{L-1}}\right]$ is the steering vector. Normally, the delays $\tau_{l}$ associated with the vector $v\left(\omega \right)$ would be identical to the delays used in D&S beamforming. For the estimation of the noise covariance matrix, the same algorithm is used as in [69], where the noise-only segments detected with a VAD are used for the computation of this matrix. In our case, we used a pre-trained enterprise-grade voice activity detector [70], which was fed with the D&S signal. For the dynamic case, the weights are time-varying, and are called $w\left(\omega ,t\right)$ because the steering vector changes over time.

We note that the impact of minimizing the signal variance (and hence the output noise power) is to impose nulls in the directional response in the direction of the L-1 noise sources of greatest power. Functionally, the difference between D&S and MVDR beamforming is that D&S beamforming is determined solely by the direction of arrival of the target. MVDR beamforming emphasizes the target in exactly the same fashion as D&S beamforming, but it suppresses the most powerful noise sources as well.

### 2.3. Self-Attentive MVDR (SA-MVDR)

This beamforming system based on deep learning is described in [44]. It has a two-stage architecture, where, first, a mask estimator based on Conv-TasNet is used to compute the instantaneous speech covariance matrix $\psi_{S}$ and the instantaneous noise covariance matrix $\psi_{N}$. In the second stage, the instantaneous covariance matrices are processed by an attentional network consisting of a stack of transformers [71] with a final layer of single-head self-attention where the attentional weights of the latter are used for the computation of speech $\Phi_{S}$ and noise $\Phi_{N}$ covariance matrices for each frame. Finally, with the covariance matrices, the beamforming weights are computed using the MVDR solution of [19], whose formulation is presented in (3):

(3) $$w\left(\omega ,t\right)=\frac{\Phi_{N}^{-1}\left(\omega ,t\right)\Phi_{S}\left(\omega ,t\right)}{{Trace(\Phi }_{N}^{-1}\left(\omega ,t\right)\Phi_{S}\left(\omega ,t\right))}\;u$$

where t represents the frame number and u is a unit vector representing the reference channel used.

**Figure 1:**
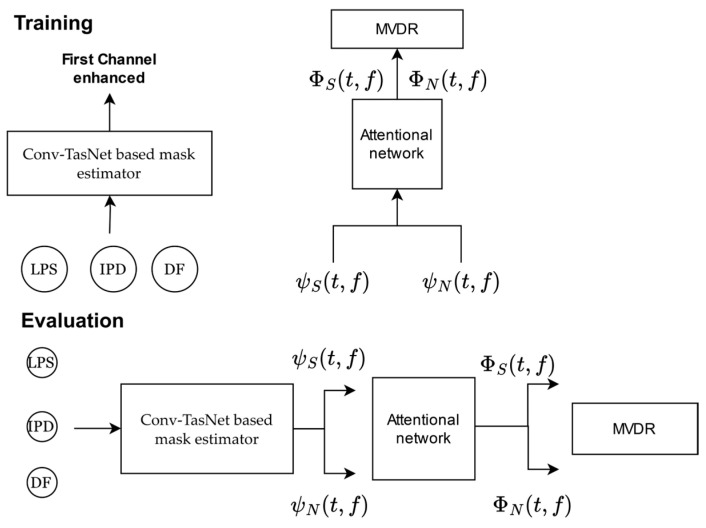
SA-MVDR training and evaluation stages.

The inputs to this architecture used in the experiments are (1) the log-power spectra (LPS) of the first channel, (2) the interaural phase differences (IPDs) between the first channel and the remaining channels (which represent the observed phase difference between the channels), and (3) the directional feature (DF) guided by the angle of arrival (AOA) [33] calculated with the steering vector obtained with the AOA registered by the PR2 robot and the computed IPDs. As mentioned before, this architecture is composed of two main blocks. The first block of the mask estimator was trained by feeding it with the three features mentioned above, as shown in Figure 1. The second block was trained with oracle masks, i.e., the speech and noise signals are known. Since the mask estimator consists of CRFs (complex ratio filters) [27], the ideal complex filter of the first channel was used for the training phase [72]. Estimating the instantaneous noise covariance matrix in the test step uses the difference between the noisy signal and the enhanced speech. Diagonal loading was performed and was used to resolve the numerical exceptions that occurred during covariance matrix inversion [73]. Additionally, the authors implemented weight smoothing because improved enhancement metrics do not necessarily lead to better speech recognition accuracy. This method smooths the weights that accumulate the statistics of the covariance matrices. For this purpose, the weights are averaged in each frame within a window of size 2L + 1 across the rows of the attention matrix (i.e., the query dimension). In this paper, SA-MVDR was evaluated with this smoothing scheme, which prioritizes ASR accuracy over enhancement metrics on a complementary basis as with SA-RNN. Figure 1 shows the SA-MVDR architecture.

The SA-MVDR beamformer showed promising results in handling moving conditions and introduced the weight smoothing mechanism. In addition, its architecture is quite flexible, easily allowing the inclusion of AOA and CRFs in the beamformer.

### 2.4. Self-Attentive RNN Beamformer (SA-RNN)

**Figure 2:**
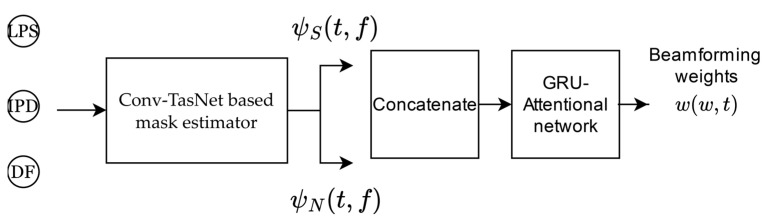
SA-RNN beamformer architecture.

The beamforming method developed in [45], consists of an end-to-end architecture based on deep learning, in which the masking process is performed by complex ratio filters (CRF) [27] using a Conv-TasNet architecture with an STFT encoder in order to estimate the instantaneous speech covariance matrix $\psi_{S}$ and the instantaneous noise covariance matrix $\psi_{N}$. The matrices are then normalized by layer normalization [74] and concatenated to be processed by a GRU [75] followed by two single-head self-attention layers [71], with the first layer operating along the temporal dimension and the second layer operating along the spatial dimension to predict the beamforming weights with a linear layer. In these experiments, the system was adapted to act in a multiple-input single-output (MISO) fashion since only the arrival angle of the speech source was used. As well as SA-MVDR, LPS, IPD and DF were used as inputs. Figure 2 is a block diagram of the architecture with inputs and outputs.

This architecture provided good performance when compared to traditional beamforming and other SOTA methods in the speaker separation task. Moreover, this architecture exploits the features of the robotic platform since it makes use of AOA. It can also cover dynamic situations by performing frame-wise processing to obtain the beamforming weights.

## 3. Experimental Procedures

### 3.1. Introduction

To address complex HRI scenarios, a robot can use multiple sensors to extract information from the environment and carry out its task. In the context of voice interaction, there are several applications that require a high-quality audio signal, for which beamforming is one of the most widely used techniques. Beamforming benefits from the robot being able to locate or track [76] multiple targets through, for example, image processing and image tracking [69], which is more robust than using acoustic information alone, especially in dynamic indoor environments. On the other hand, it is necessary that the robot, in HRI situations, be able to cover multiple environments. Beamformers based on deep learning have demonstrated promising results in speech enhancement and target separation, but they also must be trained with complex data that covers multiple situations and scenarios. There are practically no databases available for the evaluation of multichannel speech enhancement and multichannel target separation methods in an HRI context, especially for the evaluation of speech enhancement with moving sources.

**Figure 3:**
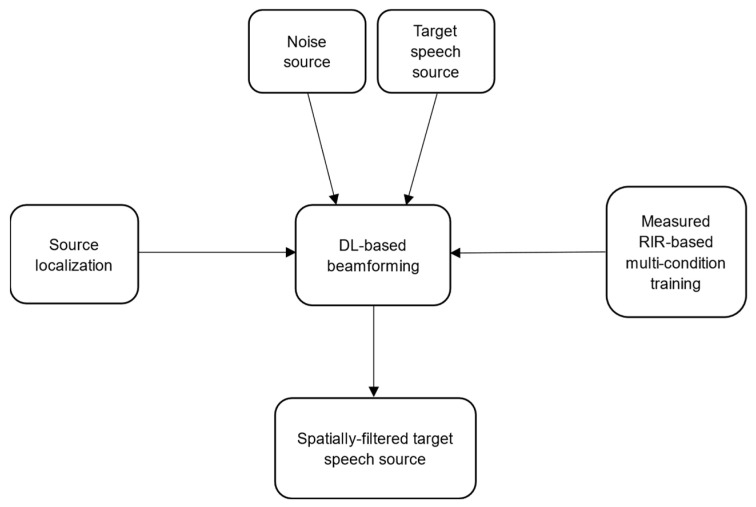
Block diagram of the signal processing in the mobile HRI platform.

Because of these considerations, successful beamforming must be informed by multiple sources of knowledge. Figure 3 shows the experimental platform used in this paper. The deep learning-based (DL) beamforming module is informed by four components: the target speech source, the noise sources, the source location, and measured-RIR-based multi-condition training. Knowledge of the angle of arrival of the target source is necessary for the beamforming to be effective; this is assumed to be accomplished using non-auditory methods not discussed in this paper. The beamformers (other than delay-and-sum or MVDR) are trained using one of three methods that are described below.

In the subsections below, we describe the overall platform we adopted, the speech and noise databases that were used, the methods used for compiling RIRs and using them to develop acoustic models, along with further specifics concerning the implementation of the deep neural networks to perform the classifications and the evaluation metrics used.

### 3.2. Mobile HRI Platform

**Figure 4:**
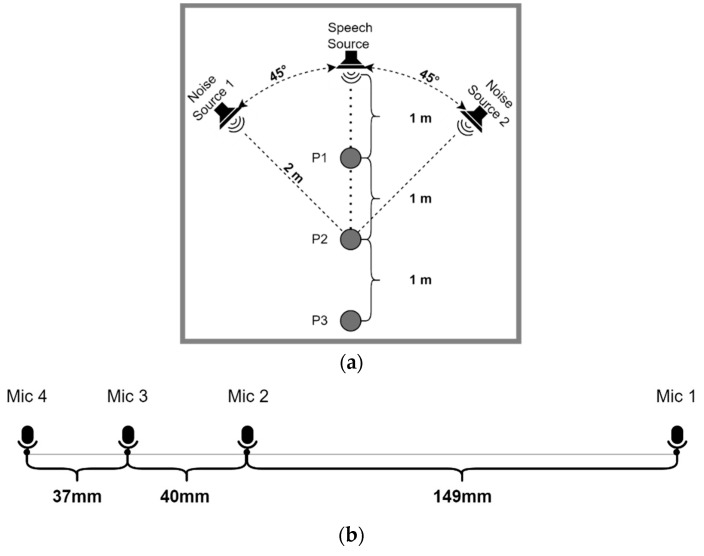

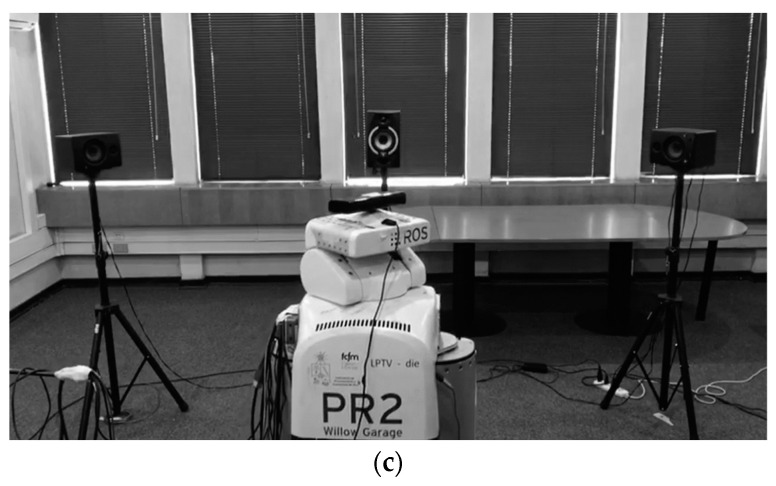
(**a**) Key dimensions used in the mobile HRI experiments; (**b**) the spacing of the microphones in the Kinect microphone array; (**c**) rear view of the HRI platform.

A state-of-the-art mobile robotic testbed was used in [67] to evaluate speech-recognition performance in indoor HRI scenarios. The platform of this testbed consists of a Personal Robot 2 equipped with a Microsoft, Redmond, WA, United States, Xbox 360 Kinect sensor, which has an array of four linear microphones mounted on its head. The PR2 robot has the ability to move and rotate its head by registering the degree of rotation, which is ideal for the development of a moving testbed. The robotic platform was deployed in a meeting room in which the locations of the speech and noise sources, as well as the positions at which measurements are made, are shown in Figure 4a. The speech source is located two meters away from position P2, and the noise sources are placed at ±45 degrees with respect to this same position. The geometry of the microphone array, with the distances of the microphones, is shown in Figure 4b, and the actual deployment of the robotic platform is shown in Figure 4c.

The testbed recorded on this platform consists of recordings of the 330 utterances of the Aurora-4 test database [77] in different scenarios, where the number of noise sources and movements present in the PR2 robot is varied.

### 3.3. HRI Databases

**Figure 5:**
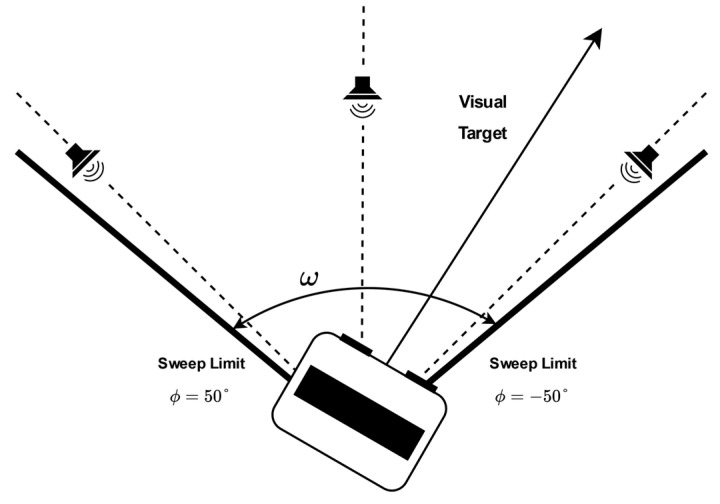
The PR2 robot’s head performs a periodic angular sweep between φ1 and φ2 at a given angular velocity for recording the testing datasets in dynamic conditions (see Table 1).

From the testbed described in Section 3.2, the databases Database-II (DBII) and Database-III (DBIII) were used. The DBII database uses only Noise Source 1, while the DBIII database uses both noise sources, as depicted in Figure 4a. These databases contain utterances recorded in both static and dynamic conditions, and, in each case, there are two sub-conditions. In the first static condition (Static 1), the robot points to the speech source, while, in the second static (Static 2), the robot points to Noise Source 1. In the Dynamic 1 sub-condition, the robot head performs a periodic angular sweep within the range of angles from –50° to 50°, as shown in Figure 5. In the Dynamic 2 sub-condition, the robot head rotates, as in Dynamic 1, and, in addition, the robot moves between positions P1 and P3, as identified in Figure 4a. Each condition consists of recording the 330 test utterances from the Aurora-4 database [77], while the restaurant noise played on the loudspeakers corresponds to the noise of the sources. The noise source volume was calibrated to obtain an SNR of 5 dB when recording speech and noise independently with the robot in position P2 at an angle of 0°. This procedure was carried out independently for DBII and DBIII. With DBIII, the volume of the noise sources was the same. Detailed database information for each condition is presented in Table 1.

### 3.4. Measuring Room Impulse Responses

We used Farina’s sine sweep method [78] to estimate the impulse responses of the acoustic channel of different actual rooms, as in our previous work [67]. In addition to the RIRs of the speech source, the RIRs of both noise sources were obtained. For the recordings, the Microsoft Xbox 360 Kinect was mounted on a tripod with a Manfrotto, Italy, Cassola, MH804-3W head, which has an angle meter with a 5° step with which the AOA of the RIRs is registered.

**Figure 6:**
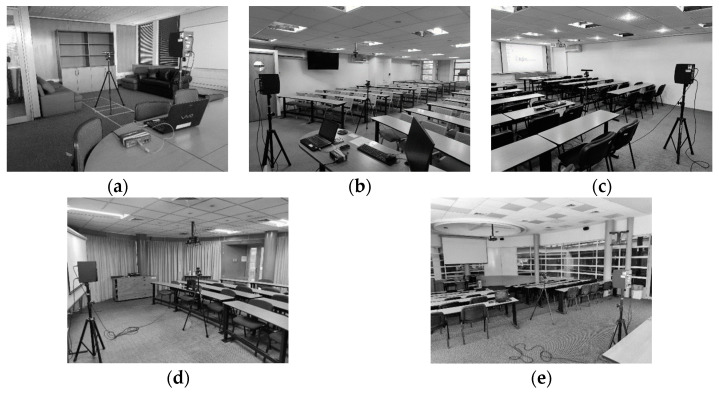
(**a**) Set up for recording RIRs: (**a**) Meeting Room; (**b**) Classroom 1; (**c**) Classroom 2; (**d**) Seminary room; (**e**) Auditorium.

The impulse responses were calculated in five rooms, which are shown in Figure 6. It can be seen that the rooms include multiple reflective surfaces, such as tables, couches, chairs or other objects, in addition to not presenting a regular geometry. In each of these rooms, 189 four-channel RIRs were obtained using the robotic platform described in Section 3.2. With this configuration, the tripod with the array of four microphones was placed in each of the three P points, recording an impulse response every 5° in a sweep from –50° to 50 (21 angular positions), a procedure that was performed for each of the three sources. The approximate dimensions and estimated values of the RT60 reverberation times are summarized in Table 2. The REW software V5.20.13 package [79] was used for the computation of the reverberation times.

### 3.5. Training the Acoustic Models

The Aurora-4 database was used to train the various acoustic models employed in this paper. This database consists of 7138 training utterances, 330 validation utterances and 330 evaluation utterances, with which three types of training sets were generated, which are differentiated by the RIRs used. The DEMAND database [80] was used to add noise, consisting of 18 different noises, of which 14 were used for training, 2 for validation and 2 for evaluation. It should be noted that each noise is recorded using a 16-microphone array, from which different channels were selected randomly during the generation of the dataset. In addition, noises produced by the PR2 robot [67] were used.

To generate each training set, 14,276 utterances were created. For 25% of these utterances, only Noise Source 1 was used, and for another 25% only Noise Source 2 was used (see Figure 4a). The remaining 50% used a noise mixture formed by both sources, with the power ratio between them ranging between –5 and +5 dB. In this case, both noise sources corresponded to the same type of noise from the DEMAND database but shifted in time. Before being added, the target speech, Noise Source 1 and Noise Source 2 are all convolved with the appropriate RIRs based on their positions, the robot head angular position and the training data set (see below). The noise from the robot is added such that the ratio of robot noise power to total power from all the noise sources (after applying the room impulse responses) is −5 to −10 db. Finally, the resulting noise was added to the speech signal with an SNR between 0 and 10 dB. This methodology was also adopted for the validation and evaluation sets, for which 1980 and 1320 utterances were created, respectively.

In all three training sets, the data were obtained by passing the original natural or degraded speech described above through measured or simulated room impulse responses (RIRs). These differ in how the RIRs and the acoustic models derived from them were obtained. The three types of training sets are as follows:

- **Measured Matched Acoustic Modeling (MMAM)**: In this type of acoustic modeling, only measured RIRs from the target meeting room are used for training, and these are recorded at locations that correspond to the actual locations of the recordings used for the HRI testing databases. These RIRs were used to generate both the training and validation set and a simulated test set.
- **Measured Multi-condition Generic Acoustic Modeling (MGAM)**: In this type of acoustic modeling, the measured RIRs from Classroom 1, the seminar room and the auditorium were used for training, and the RIRs from Classroom 2 were used for validation. In other words, the training and validation data were obtained from a multi-condition composite of indoor environments that were different from those used for the testing data. We use the acronym MGAM (for Measured Multi-condition Generic Acoustic Models) to avoid confusion between the initial “Ms” in “Multi-condition” and “Matched”.
- **Simulated Multi-condition Generic Acoustic Modeling (SGAM)**: This type of acoustic modeling is similar to the MGAM training method except that the RIRs used for training were simulated by the image source method employing the gpuRIR [55] simulator using model parameters that matched those used in the MGAM training. These RIRs were generated using the parameters shown in Table 2 from the same training and validation rooms that were used in MGAM.

### 3.6. Training the Deep Learning-Based Beamformers

The neural deep learning-based beamformers described in Section 2 were trained in a batch mode with 4-s batches using an STFT with a window size of 32 ms with 50% overlap of the frames. The training objective adopted was the scale-dependent signal-to-noise ratio loss (SNR) [81], which is defined as in (4):

(4) $$Loss=-10log\left(\frac{{\left|\left|s\right|\right|}^{2}}{{\left|\left|s-\hat{s}\right|\right|}^{2}}\right)$$

where s is the clean reverberated signal and $\hat{s}$ is the enhanced signal.

Following the notation of [41], the CRF estimator has 256 channels in the bottleneck layer, 512 channels in conv blocks, eight conv blocks in each repeat, three repeats, and the size of the CRF is 3 × 3. This configuration was used in all models in the stage of masking.

A GRU with a hidden layer size of 400 was used for the RNN in the SA-RNN model. For training, the SA-RNNN beamformer used a learning rate of 10^−4^, a gradient clipping max norm of 10 and the Adam optimization technique.

The transformer configuration of the SA-MVDR model, following the notation introduced in [71], consists of four attention heads, attention layers of dimension 256, feed-forward layers of dimension 2048 and six self-attention blocks. The CRF estimator of this model was trained with a batch size of 8, a learning rate of 10^−4^ and the Adam optimizer. The transformer was trained with a batch size of 8, a learning rate of 5 × 10^−5^, and the Adam optimizer. When evaluating the SA-MVDR, weight smoothing with window sizes of 9 and 7 were used for static and dynamic conditions, respectively. The values of the window size were chosen by a grid search evaluating sizes of L = {3,4,7,9,11} searching for the best WER in real static and dynamic conditions. This grid search was performed with SA-MVDR trained using MMAM.

The following configuration settings were considered in the early stages of the experiments. The number of repeats for the CRF estimator was varied from 1 to 4, and the CRF size was varied from 3 × 3 to 7 × 7. For the SA-RNN beamformers, the size of the hidden units was varied from 100 to 500, and the number of GRUs was either 1 or 2. For the SA-MVDR beamformer, the dimension of the feed-forward layers was varied from 1024 and 2048, and the number of self-attenuation blocks was between 2 and 6.

### 3.7. Metrics

The following five metrics were used to assess the models: (1) signal-to-distortion ratio (SDR) [82], (2) Short-Time Objective Intelligibility (STOI) [83], (3) Perceptual Evaluation of Quality (PESQ) [84], (4) Word Error Rate (WER) and (5) output Signal-to-Noise Ratio (SNR). SDR, STOI and PESQ are all standard metrics commonly used to assess speech enhancement systems. The WER was measured using the EBT-II ASR system from [67], which consists of a DNN-HMM architecture implemented using the Kaldi open-source toolkit [85]. The recipe of the ASR system is available at http://www.lptv.cl/en/hri-asr/ (accessed on 3 January 2023). SNRs were calculated using those segments where there is no speech signal as the power of the noise signal and the remaining segments as the power of the desired signal. It should be noted that this metric is only an estimate, and it should not be considered to be absolutely accurate. Nevertheless, it provides an indication of the noise reduction in real signals where it is not possible to calculate enhancement metrics due to the lack of a proper reference. For the SDR, PESQ and STOI metrics, the clean reverberated signal was used as a reference.

## 4. Results

Table 3 presents the results under the Measured Matched Acoustic Modeling test of the traditional and deep learning-based beamformers, using the PESQ, STOI, SDR, SNR and WER metrics. MMAM training was adopted for the deep learning-based beamformers.

**Figure 7:**
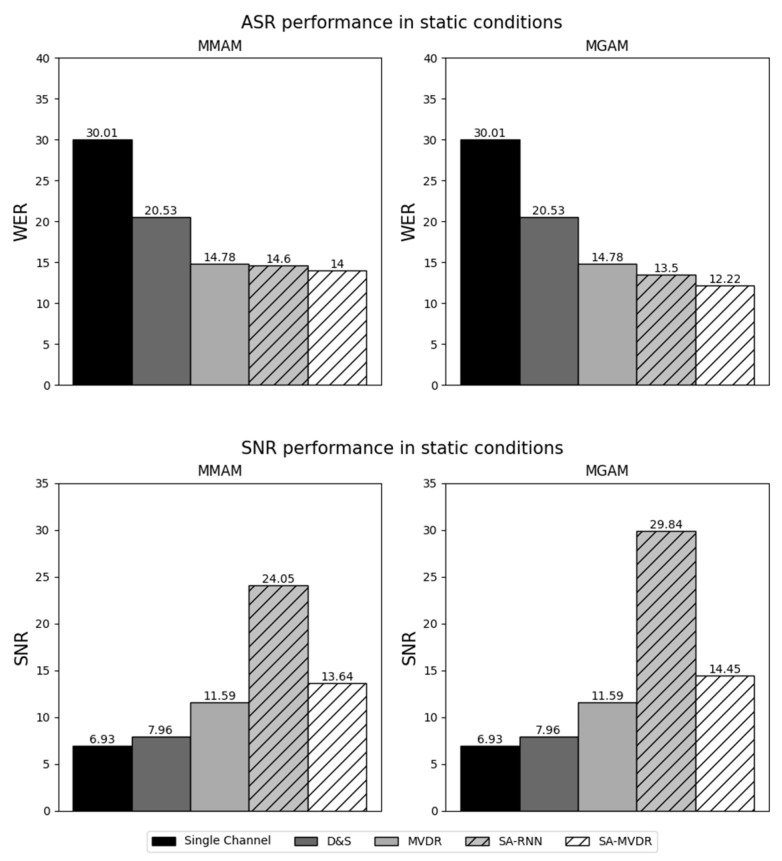
Results of Measured Matched Acoustic Modeling (MMAM) and Measured Generic Acoustic Modeling (MGAM) under static conditions.

**Figure 8:**
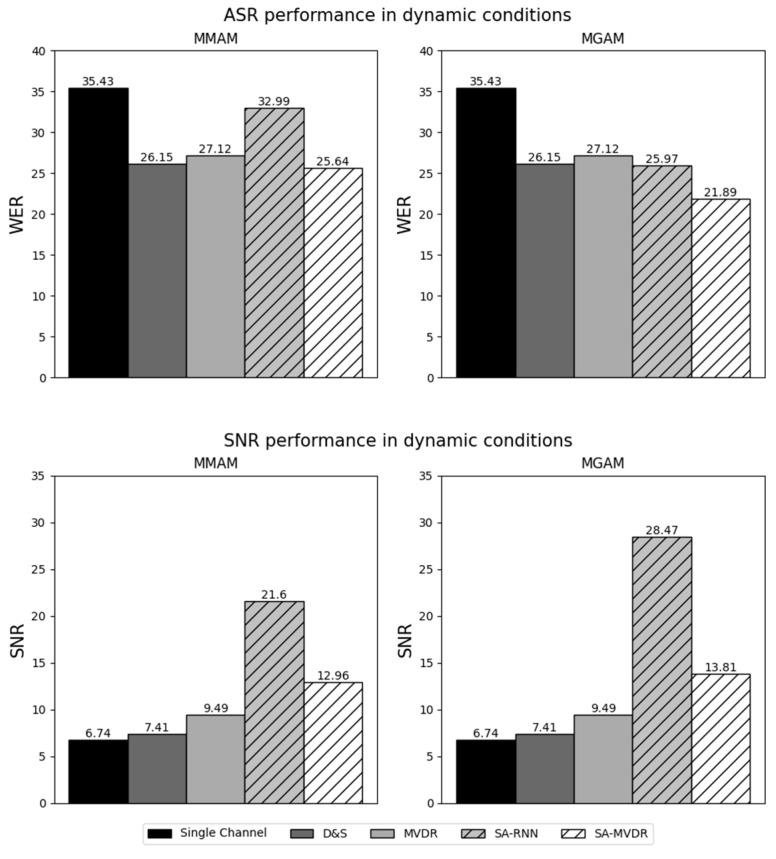
Results of Measured Matched Acoustic Modeling (MMAM) and Measured Generic Acoustic Modeling (MGAM) under dynamic conditions.

**Figure 9:**
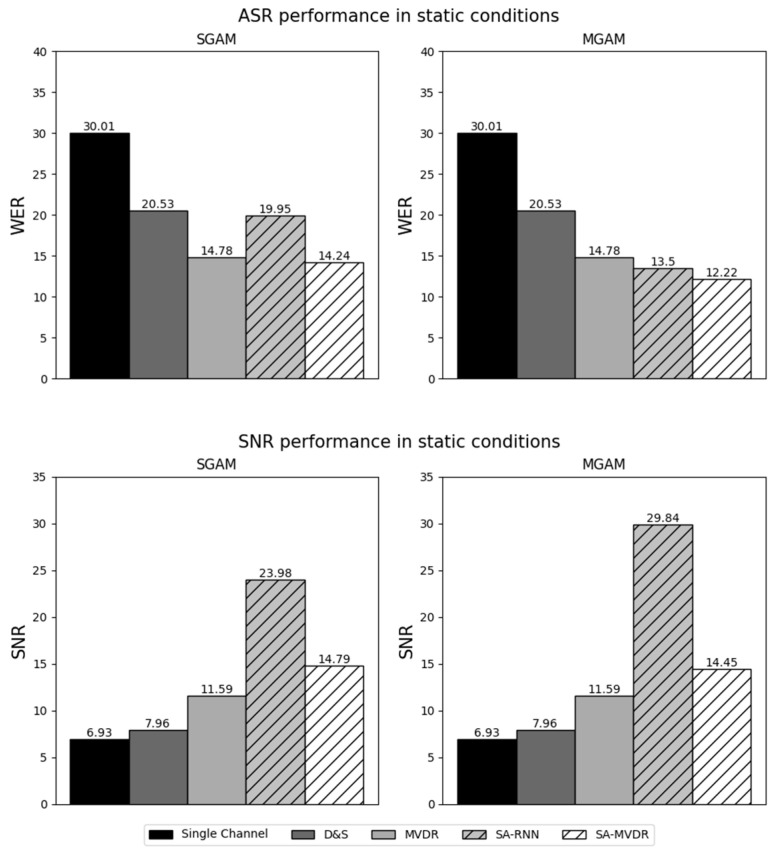
Results of Simulated Generic Acoustic Modeling (SGAM) and Measured Generic Acoustic Modeling (MGAM) under static conditions.

**Figure 10:**
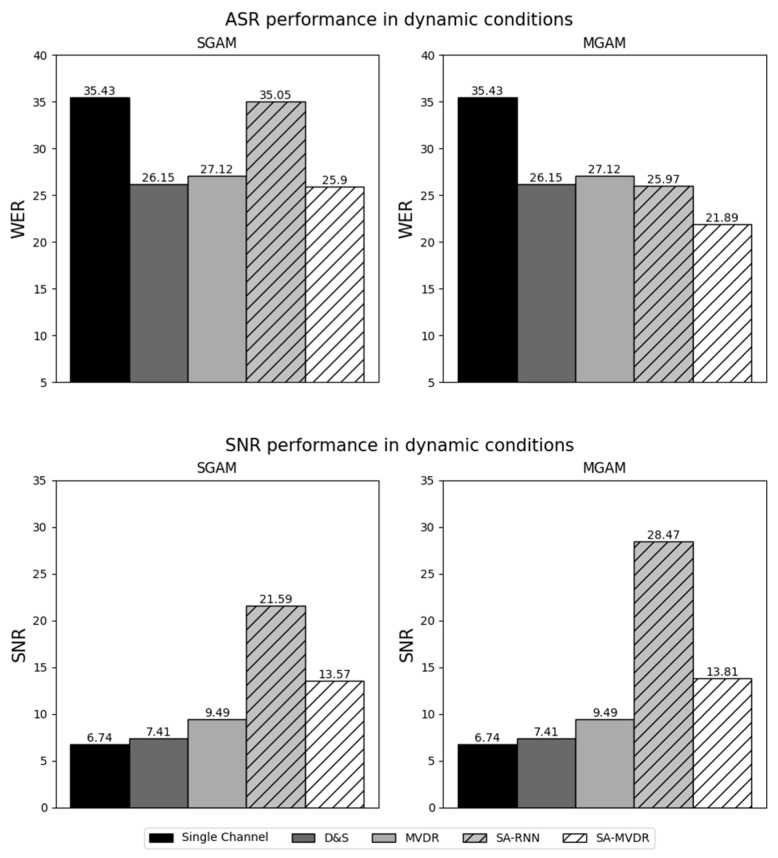
Results of Simulated Generic Acoustic Modeling (SGAM) and Measured Generic Acoustic Modeling (MGAM) under dynamic conditions.

In the figures that follow (Figure 7, Figure 8, Figure 9 and Figure 10), we present the major experimental results, first comparing results obtained with measured matched acoustic models (MMAM) to measured generic acoustic models (MGAM) in Figure 7 and Figure 8, and, subsequently, comparing measured generic acoustic models (MGAM) to simulated generic acoustic models (SGAMs) in Figure 9 and Figure 10.

In Figure 7 and Figure 8, we consider the relative effectiveness of matched training versus generic training, comparing the average WER and SNR obtained for the various beamformers using MMAM and MGAM training under static and dynamic testing conditions, respectively.

In Figure 9 and Figure 10, we consider the relative effectiveness of training using measured RIRs versus simulated RIRs, comparing the average WER and SNR obtained for the various beamformers using MGAM and SGAM training under static and dynamic testing conditions, respectively.

We provide a detailed discussion of these comparisons and their implications in Section 5, which is immediately following.

## 5. Discussion

Because this paper compares a number of different experimental conditions and because there is a relatively large number of data points tabulated in Table 3 and displayed in Figure 7, Figure 8, Figure 9 and Figure 10, we focus our discussions on a few of the observed phenomena that we consider to be of greatest importance.

### 5.1. General Comparisons of Signal Processing Techniques Considered

For all conditions, the single-microphone condition (“Single Channel”) provides the worst performance, followed by delay-and-sum beamforming (“D&S”). This is as expected, as the single-microphone conditions cannot exploit any differences in the direction of arrival for the speech and the various interfering sources. Similarly, D&S beamforming does nothing more than reinforce the signals coming from the target direction (assuming that the target direction is correctly estimated), and it does not have any knowledge of the nature or the direction of arrival of the noise sources. The MVDR, SA-RNN and SA-MVDR beamformers, on the other hand, are able to minimize the response to the various noise sources. One exception to the comments above is that D&S outperforms MVDR in WER for dynamic conditions. This is believed to have occurred because the dynamic conditions are much more challenging, and the VAD used to implement MVDR developed more errors, especially related to confusing speech with noise, which are harmful for the computation of the noise covariance matrix.

We also note that, in Figure 7, Figure 8, Figure 9 and Figure 10, results for the Channel One, D&S and MVDR microphone array conditions are the same for all training methods (MMAM, MGAM and SGAM) because these beamforming methods are not affected by which training method is used. The MVDR, SA-RNN and SA-MVDR methods generally provide better performance because they are aware of the statistics of the noise sources and, hence, can attempt to minimize the impact of the ambient noise on the signal.

Finally, we note that the SA-MVDR beamforming technique typically provides the best performance for speech recognition, while SA-RNN typically provides the best performance for speech enhancement. This remains true for all conditions considered in this study and will be addressed again below. The somewhat worse performance for SA-MVDR for the speech enhancement metrics is believed to be due to the weight smoothing mechanism, which improves speech-recognition performance at the expense of speech-enhancement performance. In the implementations we used, SA-RNN beamforming tended to produce greater degrees of distortion of the target speech signal, which caused the ASR performance to degrade. This is a reflection of the common trade-off in the speech enhancement literature that algorithms that suppress noise most aggressively and effectively also tend to introduce a greater degree of distortion to the target speech signal.

### 5.2. Impact of the Use of Matched versus Multi-Condition Generic RIRs in Training the System

The data plotted in Figure 7 (static conditions) and Figure 8 (dynamic conditions) compare the impact of training the system on data obtained by passing speech through RIRs that are matched to the testing conditions (MMAM) versus training the system using speech passed through multiple generic RIRs that are similar to, but not the same as, the testing conditions (MGAM).

For the static conditions (Figure 7), it can be seen that, when using either of the two deep learning-based beamforming techniques (SA-RNN and SA-MVDR), multi-condition generic training (MGAM) provides better performance both in terms of WER for speech recognition and SNR for speech enhancement by varying amounts. The best results were obtained using SA-MVDR for ASR (with MGAM providing a 12.9% relative decrease in WER using MGAM compared to MMAM) and using SA-RNN for enhancement (with MGAM providing an SNR that is 5.79 dB better than what is obtained using MMAM). 

MGAM outperforms MMAM even more dramatically for the dynamic conditions (Figure 8). For example, it can be seen that, using the SA-MVDR beamformer, the relative WER obtained using MGAM training is 14.6% better, and the SNR obtained for speech enhancement is 6.87 dB better. Similar results are obtained for most of the other beamformers.

These comparisons are important because they show that better results are obtained when the RIRs used to train the system are obtained from a multi-condition generic combination of relevant acoustical environments rather than the exact environment used for testing. This is good both because multi-condition training normally provides greater acoustical robustness and because matched training is frequently not possible in practical HRI scenarios. In other words, the use of MGAM training, in addition to modeling a more complex and realistic acoustic channel, allows deep learning-based beamformers to develop a greater degree of generalization to real scenarios with new acoustic conditions. In general, multi-condition training with measured RIRs is a better alternative than the use of matched HRI environments with deep learning-based beamforming techniques. It is also much less time-consuming and, hence, less expensive when a large number of speakers and scenarios must be considered. Furthermore, multi-condition generic training (MGAM) can be used to characterize situations where the dynamics of the sources change, even when the training conditions are static.

### 5.3. Impact of the Use of Measured versus Simulated Multi-Condition Generic RIRs in Training the System

The data plotted in Figure 9 (static conditions) and Figure 10 (dynamic conditions) compare the impact of training the system using multi-condition generic RIRs that are based on direct measurements of the actual room impulse responses (MGAM) versus training the system on similar multi-condition generic RIRs that are produced using a simulation of the room impulse response based on the image method [55] (SGAM).

For almost all of the conditions examined, better performance was obtained using measured RIRs (MGAM) than using simulated RIRs (SGAM). As before, the SA-MVDR beamformer provided the best WERs for the ASR experiments, while the SA-RNN provided the best SNRs for the speech enhancement experiments. The best static results were from the WER of 12.2% using SA-MVDR with MGAM training (compared to 14.2% using SGAM) and an SNR of 29.8 dB using SA-RNN with MGAM training (compared to 24.0 dB using SGAM). The differences between results for MGAM versus SGAM were even more dramatic for dynamic test conditions. The best dynamic results were the WER of 21.9% using SA-MVDR with MGAM training (compared to 25.9% using SGAM) and an SNR of 28.5 dB using SA-RNN with MGAM training (compared to 21.6 dB using SGAM).

In summary, in both static and dynamic conditions, beamforming techniques based on deep learning provide a better WER and better SNR when measured RIRs are used for training (MGAM) rather than simulated RIRs (SGAM). This behavior is to be expected as measured RIRs reflect the characteristics of the actual acoustical conditions in all their complexity, while the simulated RIRs used for SGAM are based on a simple “shoebox” model of the room.

### 5.4. Performance of Beamformers Based on Deep Learning versus Traditional Beamformers

As we have noted above, we already expect that the MVDR, SA-RNN and SA-MVDR algorithms will outperform the single-channel and D&S results because of their ability to at least partially cancel the noise signals that are incident on the microphones. Hence, the most direct comparison between traditional beamforming and deep learning-based beamformers is in comparing the results obtained using the MVDR and SA-MVDR algorithms, which are similar in principle, except that the models for the SA-MVDR processing are obtained using deep learning techniques.

In Table 3, we compare the performance of all beamforming methods using the three standard speech enhancement metrics, PESQ, STOI and SDR, along with the SNR and WER metrics that are also used in Figure 7, Figure 8, Figure 9 and Figure 10. SA-MVDR consistently outperforms MVDR, with relative score differences of 10.5% for PESQ, 16.4% for STOI, 11.6% for SDR and 24.3% for WER, and an improvement in SNR of 3.6 dB. These observations are reinforced by the results in Figure 7, Figure 8, Figure 9 and Figure 10, in which, for the static conditions, SA-MVDR provided better SNRs of 2.06 dB, 2.86 dB and 2.65 dB, and better relative WERs of 5.28%, 17.3% and 3.65% using MMAM, MGAM and SGAM training, respectively. The corresponding improvements for the dynamic conditions are even greater, with differences in SNRs of 2.05 dB, 2.86 dB and 3.2 dB, and better relative WERs of 5.46%, 19.3% and 4.50% using MMAM, MGAM, and SGAM training, respectively. These results, in which the deep learning-based SA-MVDR beamformer under simulated conditions obtains better speech enhancement and speech recognition results compared to the traditional MVDR beamformer, are consistent with expectations, as deep learning-based beamformers using masking have generally been shown in the literature to perform better than traditional beamformers under simulated conditions.

## 6. Summary and Conclusions

This paper addresses the problem of deep learning-based beamforming, with training performed using real RIRs estimated on a matched basis and on a multi-condition basis. A clear consequence of this study is that the use of multi-condition training can enable better performance for speech recognition, speech emotion recognition, prosody analysis and speech feature extraction in real static and dynamic HRI scenarios without the apparent need to record ad hoc and task-specific speech databases. The use of impulse responses measured in a multi-condition configuration is proposed to improve the performance of deep learning-based beamforming techniques for speech enhancement and robust automatic speech recognition in static and dynamic HRI contexts. We confirmed our conclusions by implementing and comparing the performance of three types of training methods for two deep learning-based beamforming techniques, trained using measured and simulated RIRs. Results were compared to results obtained using traditional beamformers.

We found that the use of deep learning-based beamformers trained in a multi-condition fashion using a combination of measured RIRs provided the best performance both for speech enhancement and speech recognition and both for static and dynamic HRI scenarios. It is encouraging that very good performance is obtained for testing conditions that are beyond the scope of the training conditions, including all evaluations involving robot motion. We found that multi-condition training using measured RIRs was more effective than similar training using simulated RIRs that were obtained using the image method. These results imply that good performance can be obtained for HRI applications in a wide variety of environments by training the system using a sufficiently large corpus of generic RIRs that are measured in multiple conditions. Further data recording or data augmentation using simulated RIRs should be neither necessary nor helpful.

We observed better generalization capability in real HRI environments using deep learning-based beamformers trained with multi-condition acoustic models that were developed using a generic set of measured RIRs rather than measurements of the actual RIRs associated with the speech in the HRI databases. Specifically, this type of modeling for training the deep learning-based beamformers provided lower WERs for speech recognition and higher SNRs for speech enhancement than training consisting of RIRs that represented the real acoustic conditions of the evaluation. Even more surprisingly, the performance increment obtained in dynamic environments using multi-condition generic models compared to models that were matched to the training data was even greater in dynamic conditions than it had been for static conditions. Based on the above, we argue that the use of training based on measured multi-condition generic RIRs allows the system to maintain good performance with more complex acoustic channels and to cover situations where the dynamics of the sources change.

This work shows that deep learning-based beamforming techniques can address real HRI situations when trained with measured multi-condition RIRs. In addition, deep learning-based beamforming outperforms conventional beamforming based on comparisons of results obtained using SA-MVDR and traditional MVDR beamformers. The improvement in performance of SA-MVDR beamformers compared to MVDR beamformers was even greater in dynamic conditions, which intrinsically are the most challenging acoustical environments.

In the future, we will explore the use of a dynamic model for database generation to further strengthen the performance of deep learning-based beamforming under dynamic conditions. We will also consider the possibility of using transfer learning techniques for these models based on measured RIRs. Studying and comparing metrics for dynamic HRI conditions are also proposed as future research.

## Figures and Tables

**Table 1:** Summary of conditions used in HRI databases.

Training Data	Condition ID	Displacement Vel. [m/s]	Angular Vel. [rad/s]	Head Angle	Ext. Noise Sources
Database-II	Static 1	0	0	0°	1
	Static 2	0	0	45°	1
	Dynamic 1	0	0.42	-	1
	Dynamic 2	0.45	0.42	-	1
Database-III	Static 1	0	0	0°	2
	Static 2	0	0	45°	2
	Dynamic 1	0	0.42	-	2
	Dynamic 2	0.45	0.42	-	2

**Table 2:** Dimensions and RT60 reverberation times of each room.

Room	Dimensions	RT60
Meeting Room	6 × 7 × 2.5	0.517
Classroom 1	7.2 × 12.5 × 2.40	0.609
Classroom 2	6.5 × 9.3 × 2.40	0.567
Seminary room	7.9 × 7 × 2.5	0.332
Auditorium	8.5 × 14 × 3.8	0.684

**Table 3:** Experimental results obtained using Measured Matched Acoustic Modeling.

Model	PESQ	STOI	SDR	SNR VAD	WER
Single Channel	2.15	0.71	4.21	6.66	33.57
D&S	2.22	0.74	5.65	7.73	23.61
MVDR	2.28	0.73	5.54	10.38	18.08
SA-RNN	3.23	0.90	12.60	22.64	12.83
SA-MVDR	2.52	0.81	6.18	13.95	13.68

## Data Availability

The raw data supporting the conclusions of this article will be made available by the authors upon request.
